# Acute, Chronic, and Treated Aortic Diseases Present Distinguishable Serum Proteome Fingerprints with Protein Profiles That Correlate with Disease Severity

**DOI:** 10.3390/biomedicines10092103

**Published:** 2022-08-28

**Authors:** Jasmin H. Shahinian, Cosima B. Hauser-Stadler, Tim Walter, Philipp Discher, Ines Derya Steenbuck, Oliver Schilling, Martin Czerny

**Affiliations:** 1 Department of Cardiovascular Surgery, University Heart Center Freiburg-Bad Krozingen, Faculty of Medicine, University of Freiburg, 79106 Freiburg, Germany; 2 Institute for Surgical Pathology, Medical Centre–University of Freiburg, Faculty of Medicine, University of Freiburg, 79106 Freiburg, Germany; 3 Department of Cardiology and Angiology, University Heart Center Freiburg-Bad Krozingen, Faculty of Medicine, University of Freiburg, 79106 Freiburg, Germany

**Keywords:** aortic pathologies, acute aortic syndrome, tissue proteomics, serum proteomics, biomarkers

## Abstract

Aortic diseases are a rare but potentially life-threatening condition. We present a serum proteomic study for a spectrum of aortic diseases including thoracic aortic aneurysms (*n* = 11), chronic dissections (*n* = 9), acute aortic dissections (*n* = 11), and surgically treated dissections (*n* = 19) as well as healthy controls (*n* = 10) and patients of coronary heart disease (*n* = 10) to represent non-aortic cardiovascular disease. In total, we identified and quantified 425 proteins across all 70 samples. The different aortic diseases represented distinguishable proteome profiles. We identified protein clusters that positively or negatively correlate with disease severity, including increase of cytosolic tissue leakage proteins and decrease of components of the coagulation and complement system. Further, we identified a serum proteome fingerprint of acute aortic dissections, consisting, among others, of enriched inflammatory markers such as C-reactive protein and members of the S100 protein family. The study underlines the applicability of serum proteomics for the investigation of aortic diseases and highlights the possibility to establish disease-specific prognostic markers.

## 1. Introduction

Aortic diseases are comparably rare with an incidence of <10 cases per 100,000 per year [1]. Yet, acute aortic syndromes are life-threatening conditions. More than 20% of patients have been reported to succumb to the disease prior to reaching a medical care center [1].

As a partial overview, aortic diseases include, amongst others, aneurysms, chronic dissections (which do not yet require surgical treatment), and acute aortic dissections. An aortic dilatation by a factor of 1.5 or greater considering the patient’s sex, age, body height and weight, is referred to as an aortic aneurysm. Dilation extent and (annual) growth define critical values for conservative, interventional or surgical treatment. In contrast, acute aortic syndromes represent a life threatening condition, in which the integrity of the aortic wall structure is severely impaired [2]. Here, acute aortic dissections (AAD) are most prevalent alongside further conditions such as intramural hematoma (IMH) or penetrating aortic ulcer (PAU). Acute aortic dissections are divided into two groups (Type A or Type B) according to Stanford classification scheme in relation to the involvement of a given aortic segment [3]. AAD typically require surgical (emergency) treatment. However, for some patients with type B aortic dissections, the disease presents sufficiently stable for a conservative, non-surgical approach [4]. In the scientific literature, dissections are referred to as “acute” within 14 days or less after dissection [5] and as “chronic” if 14 days or more have passed since dissection [6] with a refined classification distinguishing between a subacute period (15–90 days after dissection) and a chronic phase commencing 90 days after dissection [7]. Chronic dissections bear an impending risk for worsening and aortic rupture. Lifelong monitoring is required [5].

The pathophysiology of aortic diseases has remained only partially understood in current medical research [8]. Biomarkers are in the research focus with a two-fold aim: firstly, to substantiate the differential diagnosis of acute aortic syndromes in the context of rather non-specific symptoms such as chest pain; secondly as prognostic or risk stratification markers for the progression of aneurysms and chronic dissections into acute, life-threatening conditions. Serum is a rich source for non-invasive biomarkers, including proteins. Proteomics using liquid chromatography–tandem mass spectrometry (LC-MS/MS) enables the explorative, quantitative profiling of 100 s of serum or plasma proteins from only a few microliters of liquid biopsy. Previous serum proteome studies in the field of aortic diseases largely focused on comparison of selected aortic diseases vs. a set of controls [9,10,11,12]. The present study focuses on aortic pathologies including true aneurysms chronic Type B dissections and acute aortic syndromes.

At present, aortic diseases are typically diagnosed by imaging techniques. Circulating, molecular biomarkers are sought after to aid with diagnosis and to support risk stratification [13]. Here we present a serum proteome profiling study for a spectrum of aortic diseases, representing varying severity and surgical treatment.

## 2. Materials and Methods

### 2.1. Ethics

The study has been approved by the Ethics Board of the University Medical Center Freiburg (approval 342/19). Patients consented to inclusion in study.

### 2.2. Serum Sampling

Blood samples were collected in 2020 at the University Medical Centre Freiburg from patients with established diagnoses of aortic aneurysm (AA), aortic dissection (chronic or surgically stabilized, i.e., “treated) (AD), acute aortic syndrome (AAS) or coronary heart disease (CHD) patients. All 10 healthy controls were collected when presenting for allergy testing and blood work at a practice for otorhinolaryngology. The 10 adults represented the practices′ average patient composition and had, prior to inclusion, not been diagnosed with a cardiovascular disease. Up to 5 mL peripheral venous blood or, in some cases, central venous blood was collected prior to the surgical, interventional or conservative therapy scheme. Samples that tested positive for viruses such as HIV, HBV, HCV, or SARS-CoV-2 were excluded. After coagulation, the monovette was centrifuged for 10 min, 2000× *g* at 4 °C. Serum was then aliquoted and stored at −80 °C until further use.

### 2.3. Processing of Serum Samples and Measurement by Liquid Chromatography–Tandem Mass Spectrometry

The Multi Affinity Removal System (MARS), Human-14 (Agilent) was used to remove the top 14 serum proteins albumin, IgG, IgA, transferrin, haptoglobin, antitrypsin, fibrinogen, alpha 2-macroglobulin, alpha1-acid glycoprotein, IgM, apolipoproteins AI and AII, C3, and transthyretin” (manufacturer′s manual). The MARS system was used according to manufacturer′s instructions with 30 µL of serum being diluted in buffer A to a total volume of 100 µL. Following depletion, samples were stored at −80 °C until further use. The protein concentration was determined by bicinchoninic acid assay (BCA, Thermo Scientific, Dreieich, Germany). 200 μg proteome per serum sample were used for further processing. Depleted serum samples (typical volume of 600 μL after depletion) were adjusted to 50 mM for HEPES (Sigma-Aldrich) pH 7.5, 0.1% acid-labile surfactant (sodium 3-[(2-methyl-2-undecyl-1,3-dioxolan-4-yl)methoxy]-1-propanesulfonate, in-house synthesis) followed by heat denaturation (95 °C, 10 min). Disulfide bonds were reduced with 2 mM Tris(2-carboxyethyl) phosphine (TCEP, Sigma-Aldrich) at 37 °C, 10 min with orbital shaking of 600 rpm followed by blocking of free thiol groups with 20 mM iodoacetamide (IAM, Sigma-Aldrich, Taufkirchen, Germany) at 37° C, 10 min with orbital shaking of 600 rpm. Excess IAM is quenched by 10 mM dithiothreitol (DTT, Sigma-Aldrich) at 25 °C, 10 min with orbital shaking of 600 rpm. Proteins were first digested by endopeptidase Lys-C (Wako Chemicals, Neuss, Germany; MS grade) at a ratio of 1:100, 50 °C for 2 h at 600 rpm orbital shaking. Proteins were then digested by trypsin (Promega MS grade) at a ratio of 1:50, 37 °C for 18 h at 600 rpm orbital shaking. Peptides were then purified and desalted using the Phoenix Peptide Clean-up system (PreOmics) according to manufacturer’s instructions. Dried peptide samples were reconstituted in 50 µL deionized water and peptide concentration was determined by BCA. For labelling with TMTpro Mass Tags 16 plex (Thermo Scientific), the 70 samples were distributed across five label-pools. For normalization, a master-mix sample was generated, containing 5 µg of peptide from each sample. In each TMT label-pool, 2 TMT channels were used for the master-mix. 16-plex TMT labeling was performed essentially as described earlier for 10-plex TMT labeling [14]. The TMT label-pools were perfectionated by high pH reversed phase chromatography as described [14]. Liquid chromatography–tandem mass spectrometry (LC-MS/MS) analysis was performed as described [14].

### 2.4. LC-MS/MS Data Analysis

LC-MS/MS data was analyzed using MaxQuant (version 1.6.12, created by and sourced from Max Planck Institute of Biochemistry, Munich, Germany, [15]). Protein sequences are the European Bioinformatics Institute human reference proteome (without isoforms), downloaded in January 2020 and containing 20,885 entries, including additional iRT retention time normalization peptide sequences. Tryptic specificity was employed with up to 2 missed cleavages. No variable modifications were used. Cysteine carbamidomethylation and TMTpro labeling of peptide N-termine and lysine residues were set as fixed modifications. Peptide false discovery rate (FDR) was 0.05; protein FDR was 0.01. Parent Ion Fraction (PIF) was set to 0.5. Only unique peptides were used for quantitation. Proteins with one or more quantified peptide(s) were considered; however, proteins were only considered if identified and quantified in all five TMTpro label-pools. Postprocessing was performed in MSstats [16] version 3.1) using the master-mix samples to remove batch effects between the TMT label-pools. Further statistical approaches are stated at the corresponding results sections.

## 3. Results and Discussion

### 3.1. Patient Cohort

In total, 70 patients were included. The patient cohort is detailed in Table 1 and includes 7 groups with surgically stabilized dissections Type A and B taken into one group, hence 6 groups are shown in the table. The cohort size is in the range of recently published serum proteomic studies, e.g., investigating acute aortic dissection vs. acute myocardial infarction and healthy controls [11]. The cohort consists of healthy controls; patients with coronary heart disease (CHD) to represent cases with a non-aortic cardiovascular condition; thoracic aortic aneurysms, chronic type B dissections (see also additional information below), acute aortic dissections, and surgically stabilized aortic dissections (Type A and B). The chronic type B dissections did not present with life-threatening complications at the time of sampling. Hence, the chronic type B dissections in this study represent cases that were–by the time of sampling-clinically managed by regular monitoring and/or conservative measures.

### 3.2. Proteome Coverage

**Figure 1:**
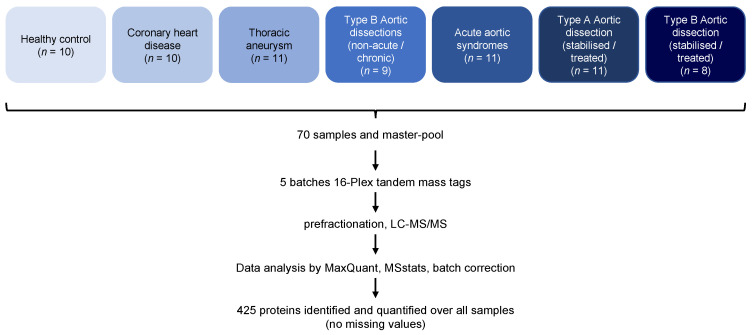
Overview of the proteomic workflow.

We employed a proteomic workflow comprising high-abundance protein depletion, TMT labeling, fractionation, and eventual analysis by LC-MS/MS (Figure 1).

**Figure 2:**
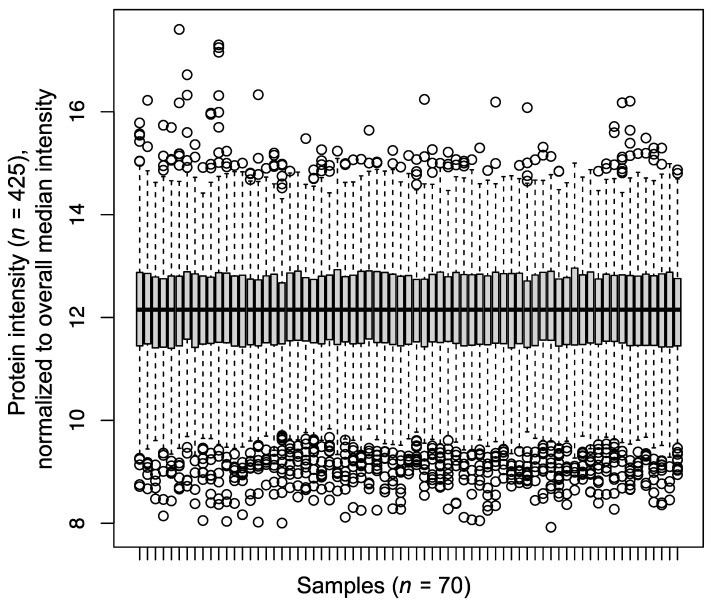
Boxplot depicting the median-normalized protein intensities for 70 serum samples of the present cohort.

In total, we identified 691 proteins by LC-MS/MS. Of these, 425 proteins were identified and quantified in each sample (*n* = 70), hence representing a “core proteome” that is devoid of missingness (Table S1). All further analyses were based on this core proteome. The proteome coverage of 425 proteins is in the range of present-day serum proteomic studies [17] with modern, fast-scanning mass spectrometers alleviating the need for high-abundance protein depletion. The “core proteome” of 425 proteins for the present study spans several orders of quantified protein intensity with a comparable range for all 70 samples (Figure 2).

### 3.3. Global Distinction of Disease Serum Proteomes by Partial Least Squares Discriminant Analysis

**Figure 3:**
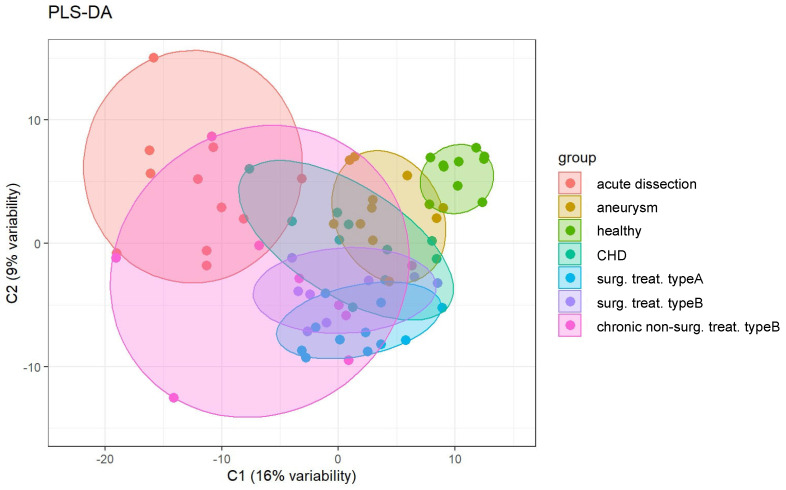
Partial least squares–discriminant analysis (PLS-DA) of the serum proteome profiles for the seven groups under investigation.

Partial least squares–discriminant analysis (PLS-DA) is a supervised statistical approach which depicts the (dis-)similarity of different conditions that are being probed by omics-type techniques. We employed PLS-DA for a global overview on the distinguishability of the serum proteomes for the seven disease/control groups (acute dissection, aneurysm, healthy, CHD, surg. treat. type A, surg. treat. type B, chronic non-surg. treat. type B) (Figure 3).

PLS-DA illustrates that the healthy control samples have the smallest intrinsic variability. Acute dissections and chronic, non-surgically treated dissections represent the largest intrinsic variability. Type A and type B surgically treated dissections largely overlap, indicating very similar proteomes. Hence, further analyses (see below) will not discriminate between these two groups. CHD serum proteome profiles show partial overlap with all groups of aortic diseases but segregate away from the healthy controls. We conclude that they represent only a poor control for heart-diseased patients with healthy aorta status. Hence, the CHD cases will be omitted from further analyses (see below). Nevertheless, this finding suggests that diverse cardiovascular diseases may yield common alterations in their serum proteomes. However, the present cohort is too small to investigate this aspect in further detail. In conclusion, PLS-DA suggests that aortic diseases present distinguishable serum proteome profiles.

### 3.4. Cluster Analysis for Proteins Whose Increase or Decrease Aligns with Disease Severity

**Figure 4:**
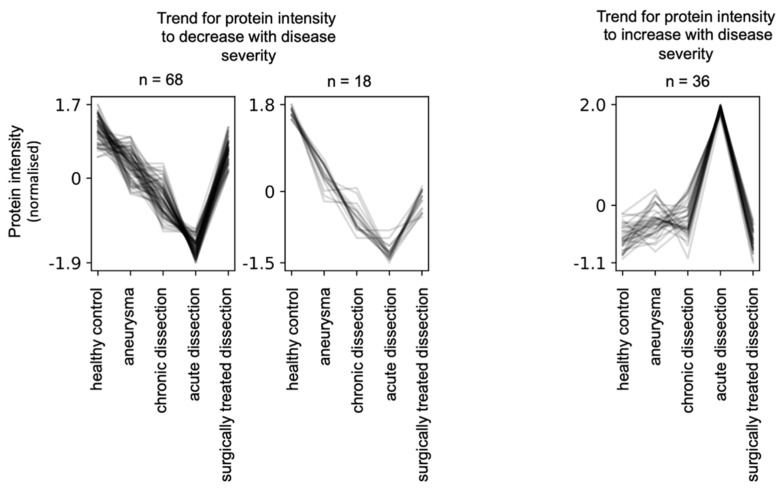
Clust analysis [18] to identify protein groups whose abundance negatively or positively correlated with disease severity together with partial reversal to healthy levels upon surgical treatment of aortic dissections. The clust algorithm determined quantile and z-score normalization as optimal.

In the present study, we sought to encompass multiple types of aortic diseases with varying severity. Hence, we aimed for a statistical approach that highlights proteins whose abundance (“intensity”) positively or negatively correlates with disease severity. To this end, we employed the clust method [18] to identify groups of proteins that have similar abundance profiles between the different disease groups. The algorithm identified five clusters, of which two are dominated by proteome alterations in acute aortic dissections (not shown). Two clusters display proteins whose abundance is negatively correlated with disease severity, alongside partial reversal to near-healthy levels upon surgical treatment (Figure 4). One cluster displays proteins whose abundance is partially positively correlated with disease severity, alongside partial reversal to near-healthy levels upon surgical treatment (Figure 4).

**Figure 5:**
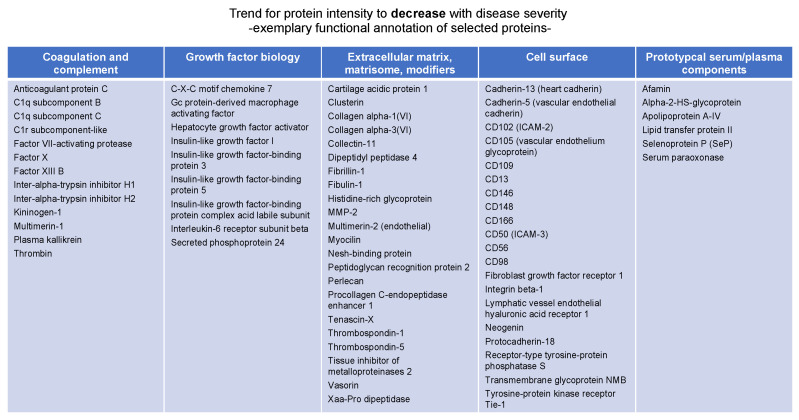
Exemplary annotation of proteins whose abundance negatively correlates with diseases severity as determined by clust analysis [18].

Manual inspection highlighted that the “negative correlation” protein group (Table S2) is largely composed of proteins involved in coagulation and complement biology, proteins of the extracellular matrix, cell surface markers, and a smaller number of prototypical serum components (Figure 5).

**Figure 6:**
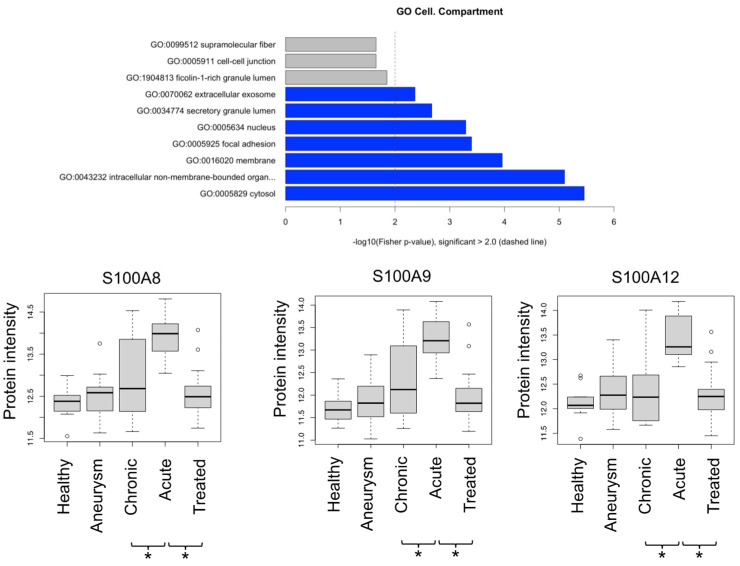
GO enrichment of proteins that are positively correlated with disease severity; depiction of protein intensities for S100-A8, -A9, -A12. * denotes *p* < 0.05 (Student *t*-test).

Manual inspection of the “positive correlation” group (Table S3) highlighted that several proteins rather presented decreased than increased levels when comparing chronic dissections with aneurysms. These proteins were manually removed, yielding a reduced list of 18 proteins for the “positive correlation” group. Gene ontology enrichment analysis using topGO highlights over-representation of cytosolic tissue leakage proteins (Figure 6). In addition, this group encompasses three members of the protein S100 family (S100-A8, -A9,-A12) (Figure 6). In a direct comparison of the different disease states using student’s t-test, they reach significantly elevated peak abundance for aortic dissections (Figure 6). S100 proteins are released inflammation [19] with A8 and A9 possibly forming a heterodimer [19]. S100 proteins may also act as endogenous damage-associated molecular patterns, hence likely being pro-inflammatory [20].

Some of our findings align with previously published serum proteomic studies on aortic diseases, despite limited comparability due to different disease spectra being investigated and differences with regard to statistical analysis. Lee et al. investigated protein profiles that are correlated with dynamics of aneurysm growth [21]. Unfortunately, it remains unclear whether serum protein levels are positively or negatively associated with aneurysm growth. Since distinction of a “decrease” and an “increase” cluster is a key characteristic of our study, we unfortunately have to refrain from integrating our findings with the Lee study. Henriksson et al. identified downregulation of bleomycin hydrolase in plasma of patients with abdominal aortic aneurysm vs. controls [22]. In our study, bleomycin hydrolase is part of the “negative correlation” cluster (Table S2), hence being in line with the Henriksson study. Burillo et al. identified paraoxonase-1 to be decreased in plasma of aortic aneurysm patients compared with controls [23,24].. In our study, paraoxonase-1 is part of the “negative correlation” cluster (Table S2), hence being in line with the Burillo study. Despite these similar outcomes, there remains noticeable divergence of with regard to published serum/plasma proteome profiles of aortic diseases with possible reasons including differences in methodology as well as differences in the patient cohort under investigation.

### 3.5. Serum Proteome Profile of Acute Aortic Dissections

The specific serum proteome signature of acute aortic dissections has been in the focus of several published studies, e.g., in comparison to healthy controls (e.g., [9,10]), acute myocardial infarction (e.g., [11]), aortic aneurysms (e.g., [12]) or combinations thereof (e.g., [12]).

**Figure 7:**
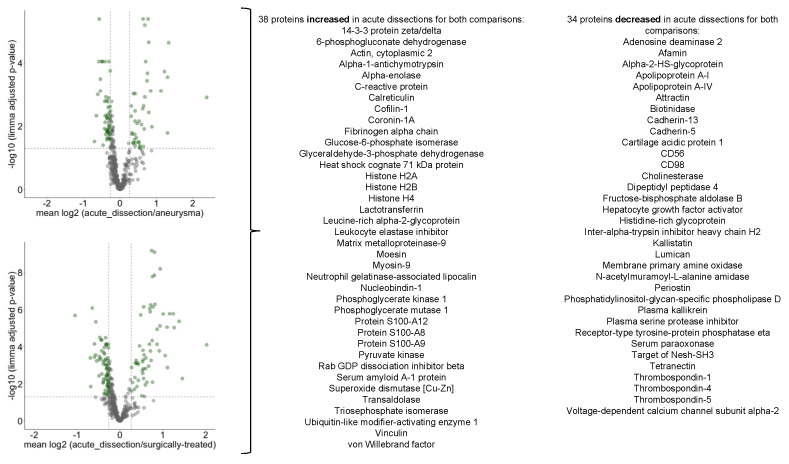
Volcano plots highlighting differentially abundant proteins for the comparison of acute dissections vs. aneurysms or surgically treated dissections.

In our study, we compared the serum proteome profile of acute aortic dissections to either aneurysms or surgically treated dissections. To this end, we employed the statistical approach of linear models of microarray analysis (limma) (Tables S4 and S5). We focused on proteins which in both comparisons met the following criteria: (1) *p*-value < 0.05 (adjusted for multiple testing) and (2) increase or decrease of abundance by at least 20%. 38 proteins were increased in acute dissections for both comparisons, 34 proteins were decreased in acute dissections for both comparisons (Figure 7). The group of increased proteins features cytosolic tissue leakage markers (e.g., glucose-6-phosphate isomerase, glyceraldehyde-3-phosphate dehydrogenase), inflammation markers (C-reactive protein, S100-A8, -A9, -A12), and matrix metalloprotease-9. The group of decreased proteins features multiple cell surface markers, components of the kallikrein-kallistatin system, and matrisomal proteins such as periostin or lumican. These functional profiles largely correspond to the previously determined protein groups that either negatively or positively correlated with disease severity, hence strengthening the analysis.

However, comparability to other studies remains limited. In addition to differences of the actual proteomic workflows, possible reasons may include different statistical approaches; for example, one study [9] employed a mere abundance level cutoff without calculation of statistical significance. Nevertheless, our results adhere to C-reactive protein and fibrinogen being discussed as marker candidates for acute aortic syndromes [25].

## 4. Conclusions and Limitations

In this study, we present the serum proteome profile for a spectrum of aortic diseases. We present candidates for protein profiles that reflect increasing disease severity and surgical treatment/repair. This approach may pave the way to establish prognostic markers, e.g., for stability of chronic dissections. At present, radiologic imaging is the diagnostic gold standard for aortic diseases. To our knowledge, there are presently no established biomarkers to distinguish different types of aortic diseases. The present study does not claim to establish such marker profiles. However, it adds to previous studies by ascertaining that aortic diseases present distinguishable serum proteome profiles. This finding may represent the foundation for future, large-scale studies to establish such biomarkers. The present study is partially limited by a comparably small sample size. Patient heterogeneity and comorbidities will be better addressed with a larger cohort size (e.g., >> 100 as spearheaded in [26]) and extension to a multi-centric study for which newly developed rapid scanning mass spectrometers might provide the required throughput [17]. Further, longitudinal serum monitoring in individual patients may unmask prognostic protein markers for increasing disease severity that may have remained masked by the pronounced inter-individual heterogeneity of serum proteome composition [26].

## Figures and Tables

**Table 1:** Description of patient cohort for the serum proteome study. CHD (coronary heart disease); BAV (bicuspid aortic valve); y (years).

	Control	CHD	Aneurysm	Chronic Type B Dissection	Acute Aortic Syndrome	Surgically Stabilised Dissections
Patients in total	10	10	11	9	11	Type A: 11 Type B: 8
Male	Anonymized upon collection, age > 21	6	9	7	5	9
Female		4	2	2	6	10
Age (years, mean ± SD)		72 ± 6	68 ± 8	66 ± 9	64 ± 16	64 ± 10
Time post aortic surgery	No surgery				Immediately prior to surgery	<6 months: 3 >6 months: 16
Aortic diameter	Not determined		<5 cm: 8 >5 cm: 3	<5 cm: 6 >5 cm: 3	<5 cm: 2 >5 cm: 9	<5 cm: 14 >5 cm: 5
Marfan	Not determined		0	0	0	1
BAV	Not determined		1	0	0	0
Hypertension	Not determined		6	4	7	18
Smoking: At present Stop < 10 y Stop > 10 y	Not determined		1 0 3	0 0 1	2 0 1	4 2 1

## Data Availability

All raw data, libraries, analysis log files, and analysis output files are available at the European Genome-phenome Archive for appropriate research use (https://ega-archive.org/EGAD00010002314 accessed on 1 July 2022). As patient-centric proteomic data is increasingly regarded as sensitive, personal data [27], EGA requires adherence to a data access agreement. The data access agreement for this dataset corresponds to the “Harmonised Data Access Agreement (hDAA) for Controlled Access Data” as brought forward by the “European standardization framework for data integration and data-driven in silico models for personalized medicine–EU-STANDS4PM”.

## Supplementary Materials

The following supporting information can be downloaded at: https://www.mdpi.com/article/10.3390/biomedicines10092103/s1; Table S1: TMTpro-based protein intensities for the 425 proteins that were identified and quantified in all 70 samples. TMTpro-based intensities are normalized to the overall median; Table S2: Proteins whose abundance is negatively correlated with disease severity;. Table S3: Proteins whose abundance is positevely correlated with disease severity; Table S4: limma statistics comparing acute dissections to aneurysms; Table S5: limma statistics comparing acute dissections to surgically treated cases.
